# Vegetation greenness and heat load drive the movements of a water-dependent, selective grazer

**DOI:** 10.1007/s00442-026-05933-2

**Published:** 2026-07-30

**Authors:** Kiara Avelyen Haylock, Francesca Parrini, Willem Maartin Strauss, Piet Beytell, Carl-Heinz Moeller, Robyn Sheila Hetem

**Affiliations:** 1 School of Animal, Plant and Environmental Sciences, University of the Witwatersrand, Johannesburg, 2050 South Africa; 2 School of Biological Sciences, University of Canterbury, Christchurch, 8140 New Zealand; 3 Applied Behavioural Ecology and Ecosystem Research Unit, Department of Environmental Science, Unisa Science Campus, Johannesburg, South Africa; 4 Ministry of Environment, Forestry and Tourism, Windhoek, 10005 Namibia; 5 African Wildlife Conservation Trust, PO Box 97401, Windhoek, Namibia

**Keywords:** Home range, Displacement distance, Hidden markov models, Behavioural trade-offs, Sable antelope

## Abstract

Low-density, selective grazers with restricted habitat requirements may be particularly vulnerable to extinction under hotter, drier conditions expected across Africa’s drylands with climate change. We evaluated how spatiotemporal variation in vegetation greenness and environmental heat load influenced movements of sable antelope, a water-dependent African grazer with selective resource requirements. We recorded hourly Global Positioning System (GPS) locations of 10 sable antelopes (*Hippotragus niger*) in Bwabwata National Park, Namibia, for up to 24 months, with concurrent hourly measurements of environmental heat load. As conditions became hotter and vegetation became browner during the dry season, home ranges increased in size and elongated towards the Kavango River. The 24-hour mean hourly displacement distance of sable antelopes increased in response to increased heat load and brown vegetation exposure, primarily due to more frequent long-distance movements to the Kavango River during the late dry season. Using Hidden Markov Models (HMMs), we identified four behavioural states of movement from GPS movement tracks: resting, foraging, local movement, and relocating. The relocating state, which was generally associated with directed movements to surface water, predominated during the late dry season. Sable antelopes increased both relocating and local movements, while decreasing foraging when exposed to high heat loads and brown vegetation. As conditions become increasingly hotter and drier, and resource availability becomes more unpredictable in space and time, species with specific resource requirements may face increased risk of extirpation, as walking farther to meet water requirements may compromise their already precarious energy balance during periods of resource limitation.

**Supplementary Information:**

The online version contains supplementary material available at 10.1007/s00442-026-05933-2.

## Introduction

Anthropogenic climate change is arguably the fastest growing threat to terrestrial biodiversity (Pereira et al. 2024). Approximately 60% of the world’s largest terrestrial herbivores (≥ 100 kg) have populations that are decreasing to the point that they are threatened with extinction (Ripple et al. 2015). Sub-Saharan Africa, where most of the large herbivore diversity occurs (Ripple et al. 2016), has warmed more rapidly than the global average and is expected to experience high temperatures unprecedented in its recent history (Engelbrecht et al. 2015). As global temperature continues to rise, sub-Saharan Africa will likely experience an increase in the frequency, intensity, and duration of droughts and heatwaves over the coming decades, as well as an increase in the occurrence of compound drought and heat events, particularly within the dryland ecosystems (IPCC 2023).

African dryland ecosystems are highly dynamic, exhibiting high spatiotemporal heterogeneity in vegetation and climate (Scholes and Archer 1997; Sankaran et al. 2005). Increasingly hot and dry conditions will impact the spatiotemporal configuration, availability, and quality of key resources for large herbivores, particularly during the dry season when food and surface water are most limited (Veldhuis et al. 2019). Behavioural flexibility, specifically the ability to adjust distributions and movement patterns, increases the capacity of large herbivores to cope with spatiotemporal variability in food resources and surface water (Mueller and Fagan 2008; Owen-Smith 2014). However, when food and water become spatially segregated, herbivores reliant on drinking water face a trade-off between staying close to permanent water sources and travelling to locate food. For example, plains zebra (*Equus quagga*) in Kruger National Park, South Africa, travelled 3–8 km every 1–2 days to drink water during the dry season (Cain et al. 2012). In the more arid Makgadikgadi, Botswana, blue wildebeest (*Connochaetes taurinus*) travelled 10–15 km every 2–4 days to drink water during the dry season (Curtin et al. 2018). These movements to distant water sources may displace other activities, such as foraging, leading to compensatory shifts in activity or trade-offs (Owen‐Smith and Goodall 2014), particularly when high temperatures in the late dry season are coupled with reduced resource availability.

Grazing herbivores are more likely to be adversely impacted by hotter and drier conditions than browsers, given their reliance on shallow-rooting seasonal grasses and greater dependence on drinking water (Western 1975; Kay 1997). Those exhibiting greater water dependence and resource selectivity, narrow habitat requirements and low local densities are predicted to be at the highest risk of extirpation (Duncan et al. 2012; Payne and Bro-Jørgensen 2020). Indeed, a number of water-dependent selective grazers, including tsessebe (*Damaliscus lunatus)* (Dunham et al. 2003, 2004), roan antelope (*Hippotragus equinus)* (Harrington et al. 1999; Havemann et al. 2016), and sable antelope (*Hippotragus niger*) (Grant and Walt 2000; Dunham 2012; Crosmary et al. 2015), have already shown population declines. These observed declines in ecologically similar species raise concerns that a multitude of environmental and ecological factors, such as habitat deterioration, resource deficiencies and enhanced predation pressure, might constrain the performance of these large herbivores across different landscapes (Ogutu and Owen-Smith 2003; Owen-Smith and Mills 2006; Owen-Smith et al. 2012a; Asner et al. 2015; Havemann et al. 2016). Given projections of increasingly hot and dry conditions, the compounding effects of thermal stress, which may exacerbate resource limitations and further challenge the persistence of these species, need to be addressed.

In this study, we assess the movement patterns and behavioural trade-offs of sable antelope, a water-dependent selective grazer currently inhabiting the hot and dry edge of its distribution, as an analogue of future conditions. We recorded hourly GPS locations of 10 sable antelopes in Bwabwata National Park, Namibia, for up to 24 months, along with vegetation greenness and environmental heat loads. We predict sable antelopes to (i) have larger home ranges during the resource-limited dry season when they are forced to search more widely for key resources, (ii) display longer 24-hour mean hourly displacement distances as vegetation greenness decreased and heat load increased, and (iii) spend a greater proportion of time in behavioural states associated with longer movements (e.g. local movement and relocating) when vegetation was brown and heat load high, at the expense of behavioural states characterised by shorter movements (e.g. foraging).

## Materials and methods

### Study area

**Fig. 1:**
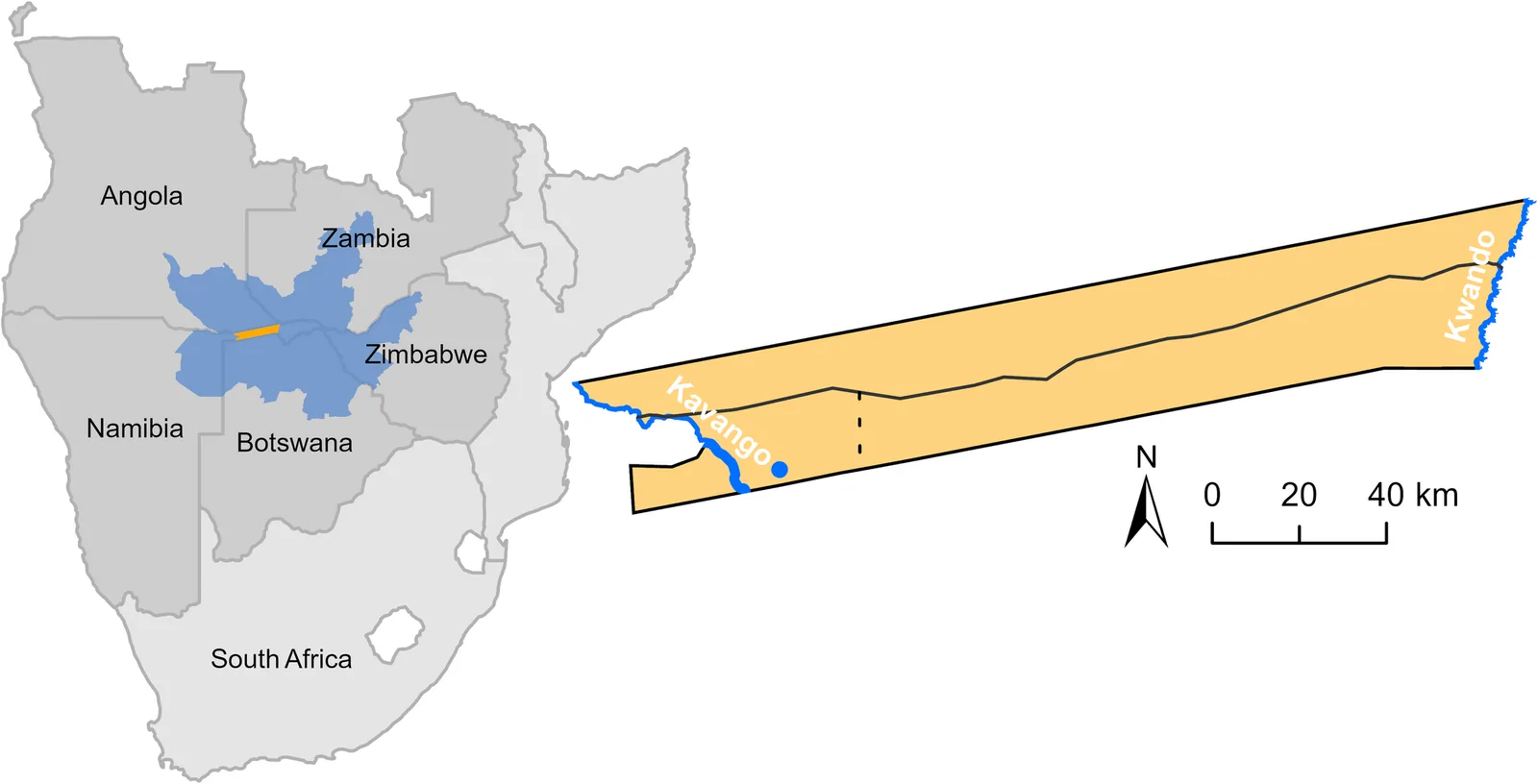
Bwabwata National Park, located in the Kavango East and Zambezi regions of north-eastern Namibia (shown in yellow), is centrally situated in the Kavango-Zambezi Transfrontier Conservation Area (blue shaded region), which spans five southern African countries (dark grey countries). The park is bordered by the Kavango River to the west and the Kwando River to the east, with Angola to the north and Botswana to the south. Sable antelopes were collared in the Buffalo Core area in the western Kavango East region of the park (area left of the stippled black line). The Buffalo Core area contains one artificial water point (location shown by a blue dot). The Trans-Caprivi Highway runs through the middle of the park (solid black line)

This study was conducted in Bwabwata National Park (17°57′S, 22°32′E), situated in the Kavango East and Zambezi regions of northeastern Namibia (Fig. 1), from 1 May 2016 to 30 April 2018. All study animals were located within or in close proximity to the Buffalo Core area in the western Kavango East region of the park (Fig. 1). The 6334 km^2^ national park extends ~ 180 km from the Kavango River on its western boundary to the Kwando River on its eastern boundary, and ~ 32 km from its northern boundary with Angola to its southern boundary with Botswana. Bwabwata National Park is centrally located in the Kavango-Zambezi Transfrontier Conservation Area, making it largely unfenced, except for standard veterinary fences along its boundary with Botswana. Bwabwata National Park is located within a summer rainfall region, with the Kavango East region receiving approximately 550–600 mm of rainfall annually, most of which occurs between November and April (Mendelsohn and Roberts 1997; Atlas of Namibia Team 2022). The dry season generally extends from April to October. Average minimum air temperatures during the coldest months (June/July) range from 6 to 8 °C, while average maximum temperatures during the hottest months (October/November) range from 34 to 36 °C (Atlas of Namibia Team 2022).

Situated in the Kalahari Basin, the park forms part of the broadleaf tree-shrub savanna biome within the miombo eco-region of southern Africa and is dominated by north-eastern Kalahari Woodland (Mendelsohn and Roberts 1997). The park consists of dry, semi-arid to open woodlands (Le Roux et al. 2009), underlain primarily by nutrient-poor aeolian Kalahari sands interspersed with clay and loam soils (Mendelsohn and Roberts 1997). The predominantly flat landscape, situated ~ 1000 m above sea level, is characterised by linear palaeo sand dunes stabilised by vegetation and ancient drainage lines (known as omurambas) in which nutrient-rich clay soils have accumulated, creating a mosaic of grassland in omurambas and woodland on dune crests (Mendelsohn and Roberts 1997; MacFarlane and Eckardt 2007). Common woody species within and surrounding Buffalo Core include *Burkea africana*, *Terminalia sericea*, *Baikiaea plurijuga* and *Acacia erioloba*, while the grass layer comprises palatable perennial species such as *Schmidtia pappophoroides*, *Digitaria eriantha*, *Panicum maximum* and *Brachiaria nigropedata*, with less palatable species including *Heteropogon contortus*, *Aristida stipitata* and *Eragrostis pallens* (Mendelsohn and Roberts 1997). In addition to the surface water provided by the bordering perennial Kavango River, the Buffalo Core area contains one artificial water point (Delta Pan). Other herbivores in the area included African elephant (*Loxodonta africana*), Cape buffalo (S*yncerus caffer*), eland (*Tragelaphus oryx*), roan antelope, tsessebe, waterbuck (*Kobus ellipsiprymnus*) and lechwe (*Kobus leche*). Predators included leopards (*Panthera pardus*), African wild dogs (*Lycaon pictus*), and African lions (*Panthera leo*), all of which occurred at low densities during the study period.

### Study animals

Ten adult female sable antelopes from separate herds were collared in April 2016, with herd sizes ranging from 11 to 48 individuals at the time of collaring. Sable antelope are large, gregarious antelope with adult cows weighing ~ 220 kg (Estes 1991). They live in female-juvenile herds with adult females, sub-adult females and youngsters forming breeding groups (Grobler 1973). Gestation spans 8–9 months with calving occurring at the end of the wet season (January to March) in southern African populations (Grobler 1973; Estes 1991). Sable antelopes typically inhabit woodland-grassland ecotones in the wooded savannas of north-eastern southern Africa (Skinner and Chimimba 2005). They are primarily selective grazers, preferring medium-height green grasses (Grobler 1981). They are considered water-dependent based on functional traits (Kihwele et al. 2020) and movements to surface water, particularly during the dry season, when they travel several kilometers every 2 to 4 days to access water (Cain et al. 2012).

An experienced veterinarian darted the sable antelope from a helicopter and fitted them with species-specific satellite tracking collars (Africa Wildlife Tracking, Pretoria, South Africa). Each dart contained a combination of thiafentanil oxalate (7 mg, Thianil, Wildlife Pharmaceuticals (Pty) Ltd, White River, South Africa) or etorphine hydrochloride (7 mg, M99, Novartis, Johannesburg, South Africa) together with ketamine (80–100 mg, Anaket-V, Bayer Animal Health Pty, Isando, South Africa) and medetomidine (2 mg, Domitor, Novartis, Johannesburg, South Africa). After collaring, the effects of thiafentanil oxalate or etorphine were reversed with naltrexone hydrochloride (75–100 mg, Trexonil, Wildlife Pharmaceuticals (Pty) Ltd, White River, South Africa) and the effects of medetomidine were reversed with atipamezole hydrochloride (10–15 mg, Antisedan, Pfizer Laboratories (Pty) Ltd, Sandton, South Africa). The collars weighed ~ 1.2 kg, less than 1% of the typical body mass of adult female sable antelopes (~ 220 kg) and were therefore unlikely to adversely affect their long-term behaviour (Stabach et al. 2020). The Animal Ethics Screening Committee of the University of the Witwatersrand approved all experimental procedures (protocol no. 2015/06/24/C). The National Commission on Research, Science and Technology (NCRST) issued the Namibian research permit (no. 2044/2015). Each collar was equipped with Very High Frequency (VHF) tracking radio transmitter and a Global Positioning System (GPS) unit recording locations at 1-hour intervals. These collars enabled periodic data transmission via satellite to a data server, allowing for near real-time downloads of the animals’ location data throughout the study period. The VHF tracking radio transmitters facilitated locating the animals to remove collars in the event of GPS communication failure. Two collars stopped functioning prematurely: one after 7 months and another after 9 months. The remaining 8 collars started failing after 12 months, with 4 of them lasting 24 months.

### Weather data

A portable weather station (HOBO Weather Logger [H21-001], Onset Computer Corporation, Massachusetts, USA) was set up at the study site to record black globe temperature (150 mm diameter matte black hollow copper sphere), and dry-bulb air temperature at hourly intervals. Black globe temperature, which integrates air temperature, wind speed, and solar radiation, provides an index of the environmental heat load experienced by animals (Hetem et al. 2007). Total monthly rainfall data for the study period were obtained from the Namibian Ministry of Environment, Forestry and Tourism from a rain gauge located within the Buffalo Core area of Bwabwata National Park.

## Data analyses

### Seasonal ranges

We delineated four seasons based on monthly variation in rainfall, Enhanced Vegetation Index (EVI), dry-bulb air temperature, and black globe temperature during the study (Table 1): early dry (May–July), late dry (August–October), early wet (November–January) and late wet (February–April). The average EVI for the study area was obtained through the Application for Extracting and Exploring Analysis Ready Samples (AppEEARS) interface (https://lpdaacsvc.cr.usgs.gov/appeears/), which provided area-averaged MODIS EVI data (MOD13A1.006; 500 m, 16-day composite) for the spatial coverage of all GPS locations throughout the study period. The EVI provides a measure of vegetation greenness that accounts for canopy background and atmospheric effects (Huete et al. 2002), with values ranging from − 1 to + 1 (negative values indicate an absence of vegetation). Temperature and EVI were averaged monthly and then across the corresponding three-month periods to derive seasonal values, which were subsequently averaged across years (Table 1). Rainfall monthly totals were summed within each season and then averaged across years (Table 1).

**Table 1:** Prevailing environmental conditions (mean ± SD) during the early dry (May–July), late dry (August–October), early wet (November–January) and late wet (February–April) seasons during which sable antelopes were free-ranging in Bwabwata National Park, Namibia (1 May 2016 to 30 April 2018)

	Early dry	Late dry	Early wet	Late wet
Total rainfall (mm)	0	0	277 ± 114	357 ± 148
Area-averaged EVI	0.257 ± 0.013	0.187 ± 0.003	0.341 ± 0.001	0.390 ± 0.005
Dry-bulb air temperature (^o^C)				
24-hour mean	18.2 ± 0.1	25.7 ± 1.0	26.9 ± 1.2	23.5 ± 0.7
24-hour minimum	7.4 ± 0.4	14.2 ± 0.1	19.3 ± 0.9	16.9 ± 0.5
24-hour maximum	30.5 ± 0.0	38.0 ± 2.6	37.2 ± 2.4	33.8 ± 2.1
Black globe temperature (^o^C)				
24-hour mean	21.6 ± 0.1	29.0 ± 0.6	30.4 ± 0.9	27.1 ± 0.3
24-hour minimum	6.7 ± 0.3	13.6 ± 0.1	18.9 ± 0.9	16.7 ± 0.7
24-hour maximum	44.0 ± 1.5	50.0 ± 0.0	51.7 ± 1.4	47.6 ± 2.8

*Notes* Values were calculated by averaging each season across years, with SD reflecting interannual variation. Area-averaged EVI (Enhanced Vegetation Index) was derived using the Application for Extracting and Exploring Analysis Ready Samples (AppEEARS) interface, providing MODIS EVI (MOD13A1.006; 500 m, 16-day composites) as an indicator of vegetation greenness across the spatial extent of all sable antelope GPS locations. Black globe temperature is indicative of environmental heat load

**Fig. 2:**
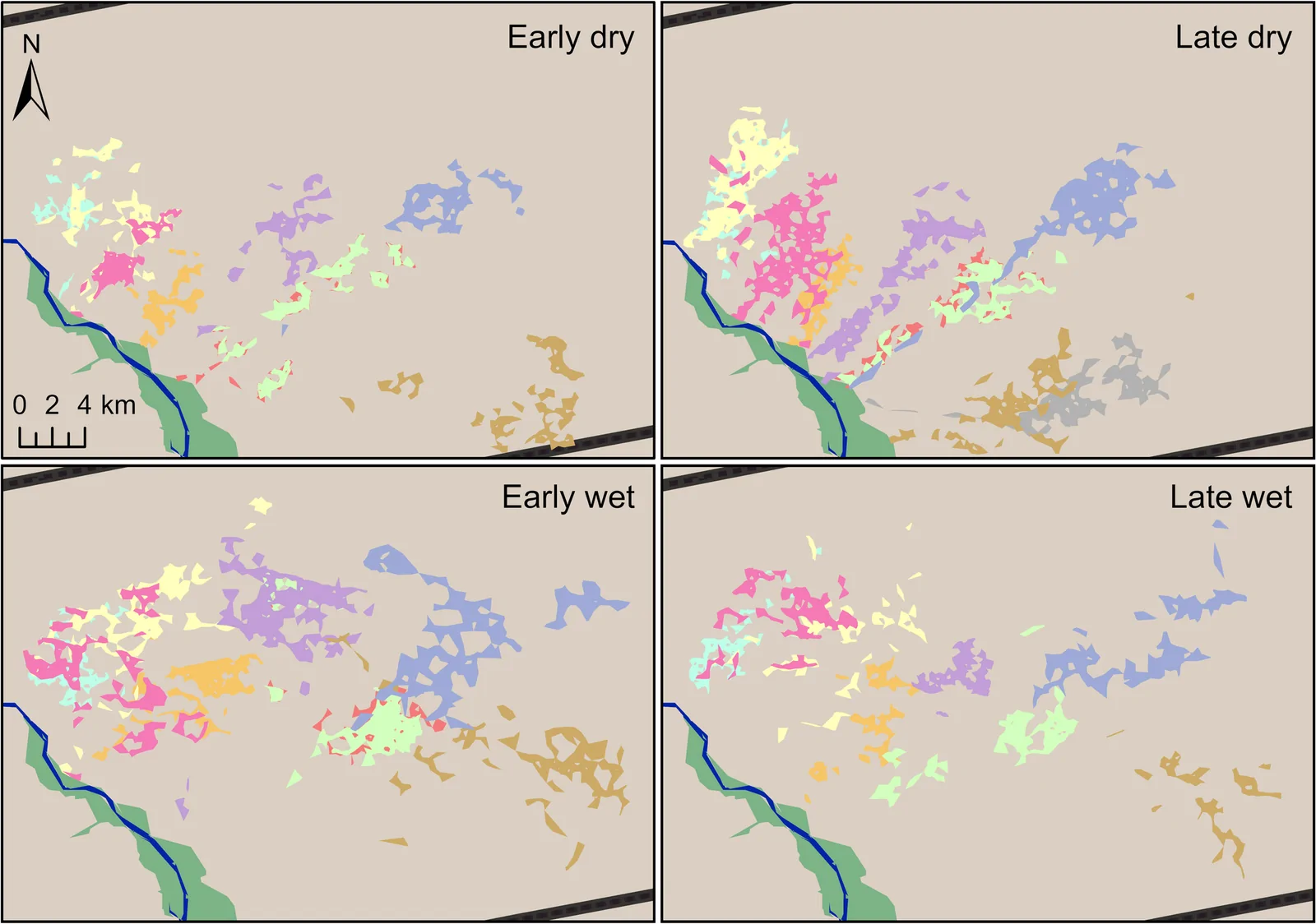
The 95% LoCoH home ranges of 10 free-ranging female sable antelopes in the Buffalo Core Area of Bwabwata National Park, Namibia, during the early dry (May–July), late dry (August–October), early wet (November–January) and late wet (February–April) season during the first year of the study (May 2016 to April 2017), for visualisation purposes when the sample size was largest (*n* = 8–10). Patterns were similar between years. Different colours represent different individuals

To investigate the variation in large-scale movement patterns, we estimated the seasonal home and core ranges of sable antelopes using the Adaptive Local Convex Hull (*a*-LoCoH) method in R (version 3.6.3, R Core Team, 2020) with the t-LoCoH package (Getz et al. 2007). The LoCoH method provides an accurate and robust home range estimation by preserving local convexity (i.e. it accounts for complex boundaries and non-convex shapes). For each individual, the adaptive kernel parameter (*a*) was selected as the minimum value that produced hulls without spurious isolates or artificial merging of unused areas due to over-smoothing, following the approach outlined by Getz et al. (2007). We defined seasonal home ranges using 95% isopleths and seasonal core ranges using 50% isopleths and calculated the extent of these ranges in ArcGIS 10.3 (ESRI 2011). For visualisation and descriptive purposes, Fig. 2 shows the seasonal home and core ranges of sable antelopes during the first year of the study (May 2016 to April 2017), when the sample size was largest (*n* = 8–10). We compared home and core range extent seasonally using separate linear mixed models (LMMs) fitted using the R package lmerTest (Kuznetsova et al. 2017). Individual identity was fitted as a random effect, and year was retained as a fixed effect to control for inter-annual variability. The response variable was log-transformed to improve normality and homoscedasticity, as assessed using residual diagnostics in the R package DHARMa (Hartig 2018). Post-hoc pairwise comparisons among seasons were conducted using Tukey-adjusted contrasts derived from the fitted LMM using the R package emmeans (Lenth 2021) (see Online Resource 1).

### Environmental variables

To interpret finer-scale movement responses to key environmental factors, we related movement data to variations in (i) vegetation greenness, assessed by a relative greenness index derived from EVI, and (ii) mean and maximum 24-hour heat load, assessed by black globe temperature. The relative greenness approach quantifies spatiotemporal variation in vegetation greenness (Relton 2015), compensating for limitations associated with soil reflection and vegetation structure (e.g. trees and shrubs vs. grasses). We estimated relative greenness using MODIS 13Q1.006 EVI time series satellite data (250 m with 16-day composites), obtained from Earth Data, Data Pool (https://lpdaac.usgs.gov/data_access/data_pool). For each GPS location, we identified the pixel in which it occurred and assigned it a relative greenness category by comparing the EVI value of that pixel at that time to the long-term mean EVI of that pixel over the 24-month study period and subsequently classified into one of three categories based on the deviation from its long-term mean and standard deviation (SD) (i.e. “brown”: EVI < mean − 0.5 SD; “average”: EVI between mean 0.5 − SD and mean + 0.5 SD; “green”: EVI > mean + 0.5 SD). For each 24-hour cycle, we then calculated the proportion of GPS locations assigned “brown” to quantify individual exposure to brown vegetation. The 24-hour mean and maximum heat load were calculated from the hourly black globe temperature recorded by the on-site weather station. A 24-hour cycle was defined as the time between consecutive sunrises. Sunrise times were obtained using the R package Stream Metabolism (Sefick 2023), which uses a sunrise function based on the National Oceanic and Atmospheric Administration (NOAA) Solar Calculator.

### Displacement distances in response to vegetation greenness and heat load

We quantified fine-scale movement as the 24-hour mean hourly displacement distance, calculated as the average straight-line distance between consecutive GPS fixes within each 24-hour cycle. To minimise bias, this calculation included only GPS locations separated by 1-hour intervals and 24-hour cycles containing 20 or more hourly locations, resulting in 4011 days (range: 54–744 days per individual) used in further analyses. We processed the GPS tracks and calculated the straight-line distances between consecutive hourly locations using the R package adehabitatLT (Calenge 2011).

To evaluate the effects of vegetation greenness and environmental heat load on 24-hour mean hourly displacement distance, we fitted linear mixed models (LMMs) using the R package lmerTest (Kuznetsova et al. 2017), with individual identity included as a random effect. The response variable was log-transformed to meet assumptions of normality and homoscedasticity, as assessed from residual diagnostics, using the R package DHARMa (Hartig 2018). Predictor variables included the proportion of brown vegetation exposure, 24-hour mean heat load, and 24-hour maximum heat load. Multicollinearity among predictors was assessed using variance inflation factors (VIFs), calculated with the R package performance (Lüdecke et al. 2021). Mean and maximum heat load are inherently related measures and exhibited moderate collinearity (VIF values > 3.5; Online Resource 1); therefore, they were not included in the same models. All predictors retained in the best supported model had VIF values of 1. The best supported model, identified by the lowest Akaike Information Criterion (AIC) using the R package AICcmodavg (Mazerolle 2023) included the proportion of brown vegetation exposure and 24-hour mean heat load (see Online Resource 1).

### Behavioural states in response to vegetation greenness and heat load

To identify the potential behavioural drivers of variation in hourly displacement distances, we fitted Hidden Markov Models (HMMs) to infer latent behavioural states from GPS tracks using the R package moveHMM (Michelot et al. 2016). For each individual, we fitted two-, three-, and four-state HMMs assuming lognormally distributed step lengths, which better captured the long-tailed data distribution compared to the gamma distribution. The four-state model provided the best model fit according to AIC (see Online Resource 1). The HMMs classified movement states based only on the probability distribution of step lengths. Including turning angle in the four-state models did not improve model fit or state classification and often resulted in biologically uninformative state assignments. This was likely the result of low turning angle concentration (κ) values for states 1 to 3, indicating limited directional information and a higher risk of overfitting. The four-state HMMs were fitted via numerical likelihood maximisation using starting values sampled from plausible ranges for each state distribution. Based on the highest maximum log-likelihood, the best model was selected from 20 iterations. The final models displayed numerical stability by converging on the same maximum log-likelihood for most of the iterations.

Classifying each movement state as a behavioural state relied on informed subjective interpretation, combining insights from the state-dependent probability distributions (Pohle et al. 2017) and previous studies of latent state allocation and 24-hour activity patterns in sable antelope (Owen-Smith et al. 2012b; Owen-Smith and Goodall 2014; Goodall et al. 2017). For this study, the four inferred behavioural states corresponded to resting, foraging, local movement and relocating. The mean step length associated with each behavioural state, averaged across all individuals, was 0.01 ± 0.003 km for resting, 0.10 ± 0.03 km for foraging, 0.41 ± 0.14 km for local movement and 1.38 ± 0.35 km for relocating.

For each individual, we calculated the proportion of time spent in each behavioural state per 24-hour cycle. This calculation included only 24-hour cycles that contained 20 or more hourly GPS locations with assigned behavioural states to reduce bias, resulting in 4011 days (range: 54–744 days per individual) for further analysis. To assess whether vegetation greenness and heat load influenced the proportion of time spent in three of the four behavioural states (foraging, local movement, and relocating), we fitted separate GLMMs for each behavioural state using a beta-binomial distribution with a logit link function. The beta-binomial distribution was selected to account for the observed overdispersion in the initial binomial GLMMs (Harrison 2015). Models were fitted in the R package glmmTMB (Brooks et al. 2017), with individual identity included as a random effect. Predictor variables included the proportion of brown vegetation exposure, 24-hour mean heat load, and 24-hour maximum heat load. Mean and maximum heat load were not included in the same models due to moderate collinearity (VIF values > 3.5). All predictors retained in the best supported models had VIF values of 1. The best supported models (lowest AIC), included the proportion of brown vegetation exposure and 24-hour mean heat load (see Online Resource 1). Model assumptions were evaluated by testing for overdispersion, zero-inflation, and residual uniformity using the R package DHARMa (Hartig 2018).

**Fig. 3:**
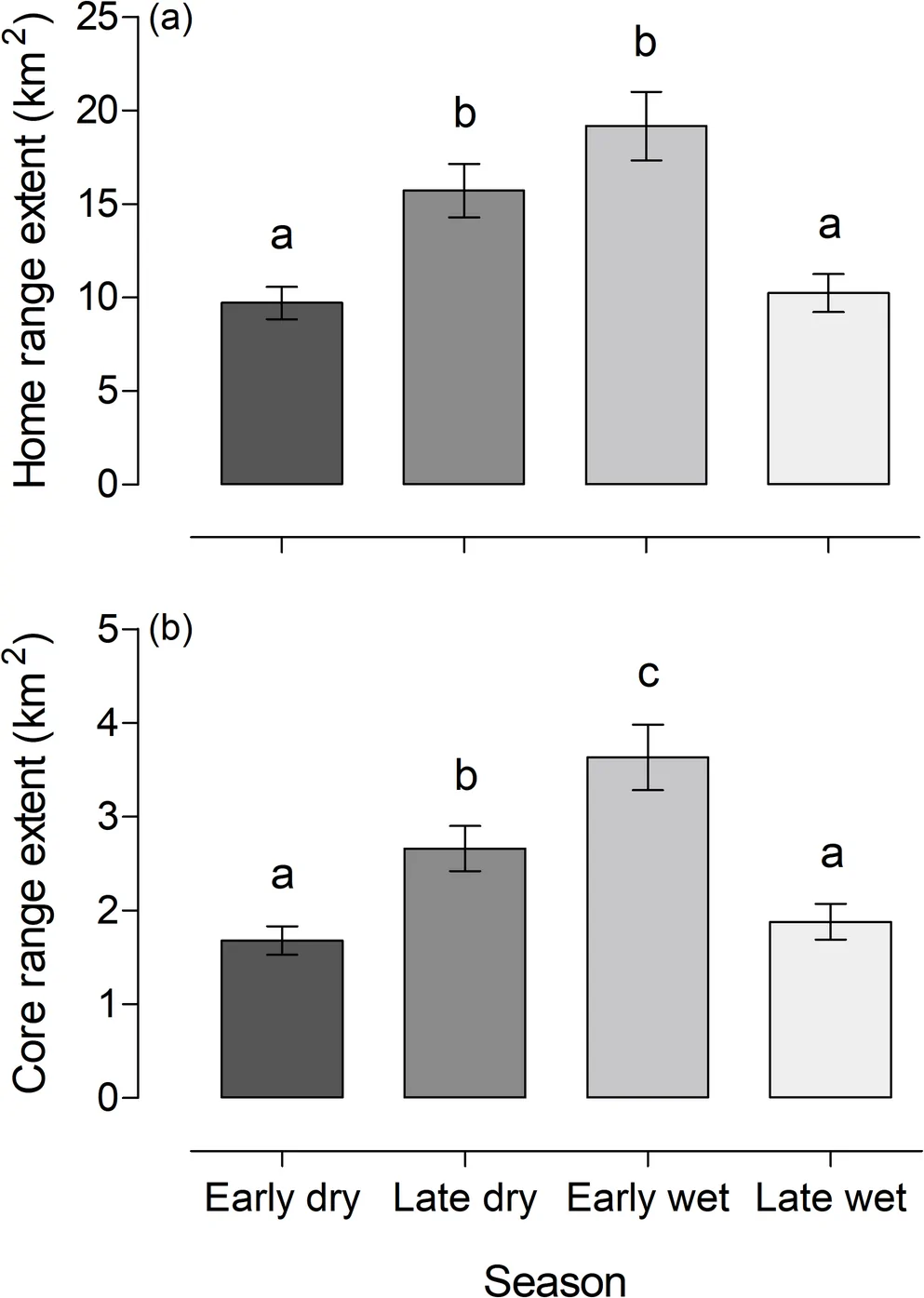
Seasonal (**a**) home (95% isopleth), and (**b**) core (50% isopleth), range sizes of free-ranging female sable antelopes across the early dry (May–July), late dry (August–October), early wet (November–January) and late wet (February–April) seasons during the study period (1 May 2016–30 April 2018). Seasonal ranges were estimated using the *a*-LoCoH method. Values are estimated marginal means (± SE) from linear mixed models, marginalised over year which was retained as a control covariate. Different letters above bars indicate statistically significant differences (*P* < 0.05; see Online Resource 1) based on Tukey-adjusted pairwise comparisons

**Fig. 4:**
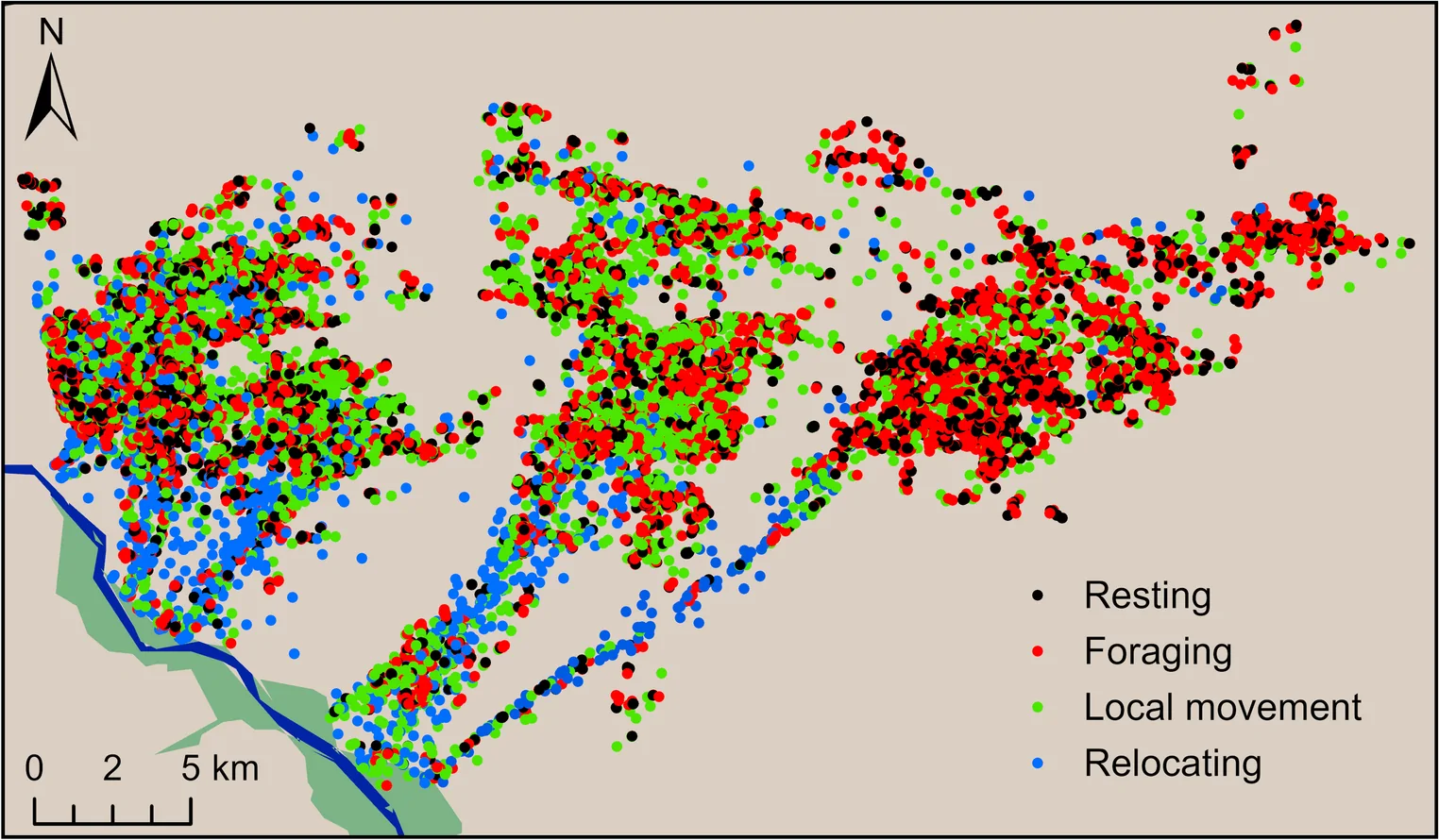
The behavioural state allocation relative to GPS locations of three representative free-ranging female sable antelopes in Bwabwata National Park, Namibia, from 1 May 2016 to 30 April 2018. Four-state Hidden Markov Models (HMMs) were used to infer behavioural states from movement tracks with inferred states corresponding to resting, foraging, local movement and relocating. Long, relocating movements (blue) were mostly directed to and from the Kavango River with the progression of the late dry season

**Fig. 5:**
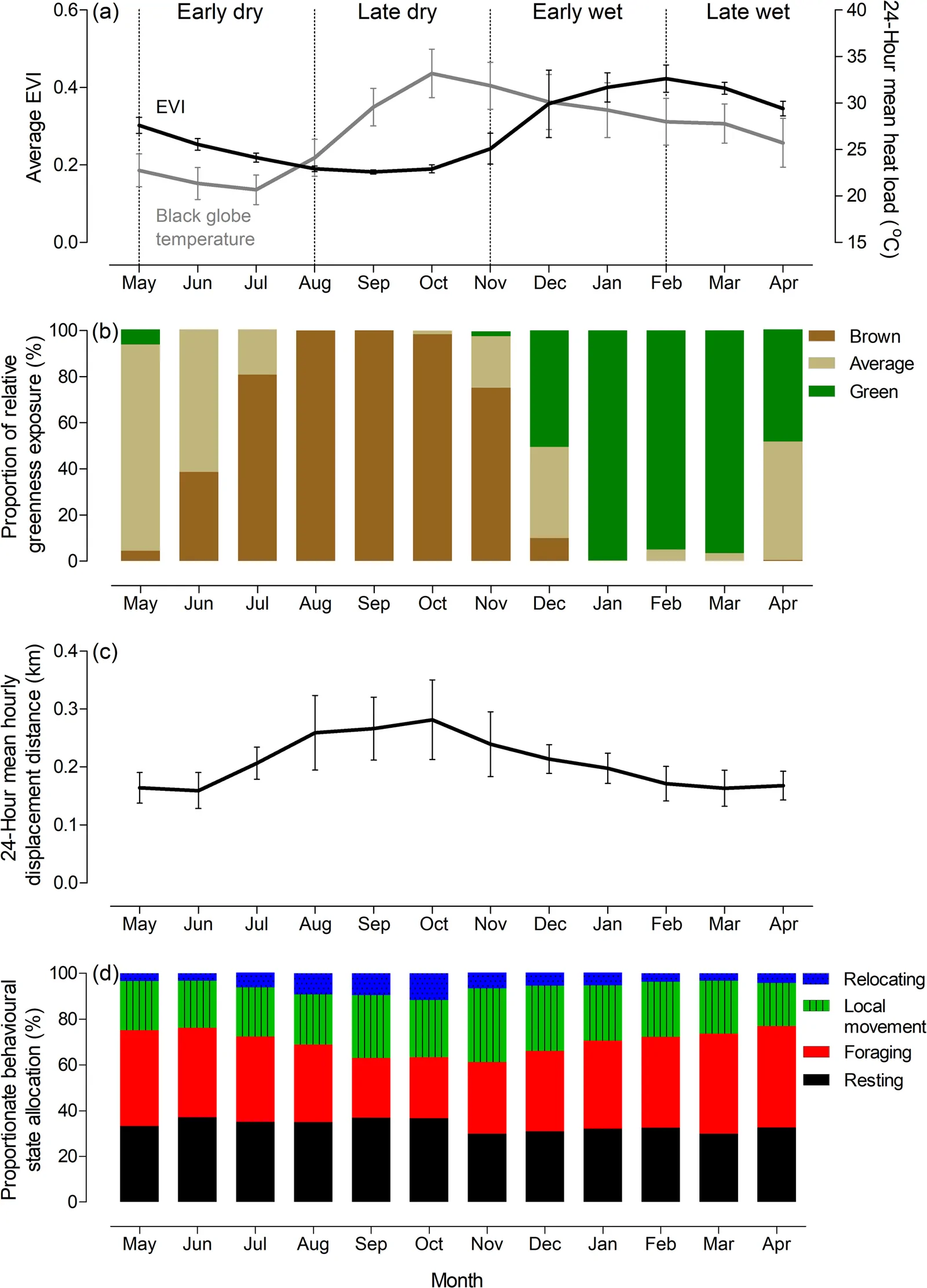
Monthly variation (mean ± SD) in (**a**) area-averaged EVI (black line) and mean 24-hour heat load (black globe temperature; grey line), (**b**) proportion of time spent in three relative greenness categories per 24-hour cycle by sable antelopes, (**c**) mean hourly displacement distance per 24-hour cycle, and (**d**) proportion of time per 24-hour cycle spent in behavioural states of resting, foraging, local movement and relocating, across the study period (1 May 2016–30 April 2018). Monthly means of area-averaged EVI and heat load are pooled across years. Monthly means for movement and relative greenness variables were calculated by averaging daily values within each individual for each month (pooling across years), and then averaging across individuals. A 24-hour cycle was defined as the period between consecutive sunrise times

Unless otherwise stated, all data processing and statistical analyses were conducted in R (version 4.5.0, R Core Team, 2025). Maps (Figs. 1, 2 and 4) were produced in ArcGIS Pro (version 3.3.0, ESRI Inc 2024; Basemap: OpenStreetMap https://www.openstreetmap.org/copyright). Graphs (Figs. 3 and 5) were produced in GraphPad Prism (version 5.02, GraphPad Software, Boston, Massachusetts, USA).

## Results

### Seasonal ranges

The sable antelopes in our study occupied adjacent seasonal home ranges in the western half of Bwabwata National Park, near the Kavango River, with seasonal variations in home range locations, shapes, and sizes (Fig. 2) that did not differ across years (year effect: β = -0.101, 95% CI = -0.249, 0.041, t = -1.36, *P* = 0.18). Home ranges were smallest during the early dry season (range: 7.3–19.8 km^2^; Fig. 3a), when environmental heat load was lowest, and vegetation greenness (EVI) was low with no rainfall (Table 1). As the dry season progressed, heat loads increased and vegetation greenness further decreased (Table 1), as home ranges elongated towards the Kavango River (Fig. 2) and increased in size by ~ 54% on average from the early dry to the late dry season (range: 11.9–24.2 km^2^; Fig. 3a). With the onset of rainfall and a substantial increase in vegetation greenness in the early wet season (Table 1), home ranges retracted from the Kavango River (Fig. 2) but remained large (Fig. 3a). As the wet season progressed, vegetation greenness remained high while heat loads decreased (Table 1), and home ranges remained distant from the Kavango River (Fig. 2) but decreased by ~ 44% in size from the early wet (range: 13.1–33.5 km^2^) to the late wet season (range: 7.3–19.3 km^2^; Fig. 3a). The extent of the late wet season range was similar to that of the early dry season (Fig. 3a). Like the seasonal changes in home range extent, core range extent did not differ across years (year effect: β = -0.124, 95% CI = -0.271, 0.019, t = -1.65, *P* = 0.11) but differed seasonally. Core ranges were small during the early dry season (range: 1.3–2.8 km^2^), increased by ~ 31% during the late dry season (range: 1.7–4.2 km^2^), further expanding into the early wet season (range: 2.4–5.9 km^2^), and then decreased by ~ 46% in extent during the late wet season (range: 1.3–3.5 km^2^; Fig. 3b).

### 24-Hour mean hourly displacement distance

As the dry season progressed from May to October, sable antelopes progressively increased their 24-hour mean hourly displacement distance (Fig. 5c). This increase corresponded with rising environmental heat loads (Fig. 5a) and increased exposure to brown vegetation (Fig. 5b). By the end of the late dry season (October), when conditions were at their hottest and brownest (Fig. 5a), sable antelopes were moving substantially further per hour per day compared to the start of the dry season (May), with mean hourly displacement increasing by ~ 1.7 times (~ 118 m) (Fig. 5c), before declining again during the wet season months. The positive association between the 24-hour mean hourly displacement distances and increased heat loads and exposure to brown vegetation (Table 2) confirmed our observation that sable antelopes walked greater distances in hotter and drier conditions. The sable antelope located farthest from the river displayed an average round-trip distance of 24.8 ± 5.3 km (range: 12.7–30.1 km) approximately every 5 days (range: 4–6 days) to drink water from the Kavango River during the late dry season (August to October).

**Table 2:** Linear mixed model results for the relationship between the average hourly distance (km) moved by free-ranging female sable antelopes per 24-hour cycle and environmental variables, namely the proportion of time exposed to brown vegetation and 24-hour mean heat load (mean black globe temperature, °C), prevalent in Bwabwata National Park between 1 May 2016 and 30 April 2018

	β	95% CI	t	*P*
**24-Hour mean hourly displacement distance**				
Proportion of brown vegetation exposure	0.231	0.193, 0.270	11.79	< 0.0001
24-hour mean heat load	0.020	0.016, 0.024	10.44	< 0.0001

*Model descriptions* log (24-hour mean hourly displacement distance) ~ proportion of brown vegetation exposure + 24-hour mean heat load + (1 ǀ individual identity), *n* = 10, *N* = 4014. Model estimates (β) and 95% profile confidence intervals (95% CI) are presented on the log-transformed scale. The proportion variable used as input in the models ranged from 0 to 1. A 24-hour cycle was defined as the period between consecutive sunrise times

### Behavioural states

As illustrated for three representative individuals (Fig. 4), GPS locations classified as foraging behaviour were generally clustered, whereas those classified as relocating behaviour were mostly associated with widespread movements, particularly directed movements towards the Kavango River, but possibly also movements to other foraging areas. As heat loads increased (Fig. 5a) and exposure to brown vegetation increased (Fig. 5b), sable antelopes increasingly spent more time per day in a relocating behavioural state, spending ~ 3.5 times more time relocating by the end of the dry season (~ 2 h more per day, Fig. 5d). Conversely, as exposure to high heat load and brown vegetation increased with the progression of the dry season, sable antelopes spent  ~ 1.6 times less time foraging by the late dry season (~ 3.5 h less per day) (Fig. 5d). This pattern was confirmed by the GLMMs, showing that increased 24-hour mean black globe temperature and increased proportion of brown vegetation exposure were associated with an overall increase in relocating behaviour and a decrease in foraging behaviour (Table 3). Sable antelopes also displayed an increase in the proportion of time spent in local movement with increased exposure to brown vegetation and high 24-hour mean black globe temperature (Table 3), spending  ~ 1.2 times more time (~ 0.8 h per day) in local movement by the end of the dry season compared to the start of the dry season (Fig. 5d).

**Table 3:** Beta-binomial generalised linear mixed model results for the relationship between the proportion of time that free-ranging female sable antelopes spent in three behavioural states (relocating, foraging, local movements) per 24-hour cycle, and environmental variables, namely the proportion of time exposed to brown vegetation and 24-hour mean heat load (mean black globe temperature, °C), prevailing in Bwabwata National Park between 1 May 2016 and 30 April 2018

	β	95% CI	z	*P*
**Proportion of 24-hour cycle in relocating state**				
Proportion of brown vegetation exposure	0.565	0.461, 0.670	10.58	< 0.0001
24-hour mean heat load	0.037	0.027, 0.047	7.29	< 0.0001
**Proportion of 24-hour cycle in foraging state**				
Proportion of brown vegetation exposure	-0.426	-0.466, -0.386	-20.78	< 0.0001
24-hour mean heat load	-0.029	-0.033, -0.025	-14.19	< 0.0001
**Proportion of 24-hour cycle in local movement state**				
Proportion of brown vegetation exposure	0.145	0.095, 0.194	5.76	< 0.0001
24-hour mean heat load	0.023	0.018, 0.028	9.50	< 0.0001

*Model descriptions* proportion of 24-hour cycle spent in a behavioural state ~ proportion of brown vegetation exposure + 24-hour mean heat load + (1 ǀ individual identity), *n* = 10, *N* = 4014, fitted using a logit link function. Model estimates (β) and 95% profile confidence intervals (95% CI) are presented on the logit (link) scale. The proportion variables used as input in the models ranged from 0 to 1. A 24-hour cycle was defined as the period between consecutive sunrise times

## Discussion

We investigated the movement patterns of sable antelope, a water-dependent, resource-specialist antelope in response to resource limitations under high heat load. To our knowledge, we have documented the longest directed movements to and from water within a single day by a non-migratory, water-dependent selective grazer, with one sable antelope covering an average round-trip distance of ~ 25 km approximately every five days during the hot, late dry season. These long, directed movements to the Kavango River, were associated with the combination of high environmental heat load, decreased ephemeral surface water and low water content in browning forage, predominantly occurred during the late dry season when sable antelopes likely relied more on evaporative cooling to dissipate heat in the hot and dry conditions. The additional time spent travelling to water came at the expense of time spent foraging when grass availability and nutritional value were most limited. By evaluating movement patterns in relation to prevailing vegetation greenness and heat load, we were able to quantitatively confirm low vegetation greenness and high heat load as drivers of the increased directed movement in sable antelopes facing resource limitations.

Our estimates of home range sizes are within the range of estimates previously reported for sable antelopes, which vary widely from 2.4 km^2^ to 118 km^2^ (Grobler 1973; Wilson and Hirst 1977; Sekulic 1981; Owen-Smith and Cain 2007; Hensman et al. 2014a; Mangueze et al. 2026). Many of these estimates were calculated using minimum convex polygons (MCP), which may overestimate the home range size by 50–60% compared with methods like KDE and LoCoH (Owen-Smith and Cain 2007; Hensman et al. 2014a). The LoCoH home range and core range estimates of our ten sable antelopes were mostly congruent with the LoCoH range estimates of three adjacent sable antelope herds in the Kwedi area of the nearby Okavango Delta, Botswana (Hensman et al. 2014a). However, in some instances, sable antelopes in our study displayed home ranges up to 7 km^2^ larger during the dry season and 13 km^2^ larger during the wet season than sable antelopes in the Kwedi. Home range occupation and movement patterns of sable antelope are inherently dependent on the local ecological conditions experienced by individuals or herds (Owen-Smith et al. 2010) and can be influenced by habitat composition, food availability, surface water distribution, herd density, competition or predation risk (Owen-Smith and Cain 2007; Rahimi and Owen-Smith 2007; Cain et al. 2012; Chirima et al. 2012; Macandza et al. 2012a, b; Hensman et al. 2014a; Mangueze et al. 2026). Because of a relatively small sample size (*n* = 10 individuals), we did not have the statistical power to analyse these covariates.

The observed increase in home range size in sable antelope with progression of the dry season and into the early wet season likely reflects spatial segregation of resources (Mueller and Fagan 2008; Owen-Smith et al. 2010). Indeed, some selective grazers expand their ranges during the dry season when forage and surface water become increasingly segregated, but show little seasonal variation in range extent where resources remain readily available and accessible (Wilson and Hirst 1977; Henley 2005; Owen-Smith and Cain 2007; Rahimi and Owen-Smith 2007; Cain et al. 2012; Havemann et al. 2022; Muposhi et al. 2025; Mangueze et al. 2026). Depletion of high-quality resources under intense grazing competition in mega-herds of migratory wildebeest in the Serengeti resulted in greater wet season displacement and space use (Hopcraft et al. 2014), a mechanism that differs from the resource-tracking behaviour likely shaping early wet season space use in the smaller, resident sable antelope herds in our study. The large and widespread home ranges during the early wet season may indicate that sable antelopes were attempting to increase their intake of high-quality grass (i.e. crude protein) by following patchy post-rain grass green-up to meet their increased nutritional requirements during late gestation and parturition (November to March) (Gittleman and Thompson 1988). Specific mineral requirements during reproduction may have further enhanced functional heterogeneity across the landscape, with Mopane woodlands providing sodium-rich drinking water (Vittoz et al. 2020) for roan antelope, a closely related selective grazer, in northern Botswana (Havemann et al. 2022). Similar spatial segregation of mineral-rich soils in Bwabwata National Park (Mendelsohn and Roberts 1997) may have increased the space use of sable antelope during the early wet season.

As the wet season progressed and resource availability increased, sable antelopes constricted their home ranges. Narrowing of home ranges during the late wet and early dry seasons may reflect the focused foraging efforts of sable antelope in areas retaining green leaf forage, such as along the omuramba grasslands or within strips of woodland on dune crests in our study area. Sable antelopes preferentially select green grass during the dry season (Macandza et al. 2012a; Hensman et al. 2014b; Owen-Smith et al. 2013), including green grass persisting in vlei areas (Parrini and Owen-Smith 2010) or green regrowth in recently burned areas (Magome et al. 2008; Parrini and Owen-Smith 2010). Access to pre-formed water from green forage, together with free surface water from ephemeral pools on-site (Naidoo et al. 2020), likely enabled sable antelopes to occupy ranges up to 20 km from the Kavango River during the wet season (Fig. 2).

Interspecific interactions likely prevented the sable antelope in our study from occupying home ranges adjacent to the river since competitive exclusion (Macandza et al. 2012a, b) and increased predation risk (Harrington et al. 1999; Chirima et al. 2012) from more abundant water-dependent grazers often forces rarer antelope species to forage or occupy home ranges further away from water sources, even during resource-limited periods (Cain et al. 2012; Nauyoma et al. 2025). Both sable antelope (Hensman et al. 2014a) and congeneric roan antelope (Havemann et al. 2022) in northern Botswana occupied dryland sandveld-woodland regions further away from permanent water during the dry season, while avoiding the floodplain exploited by more common water-dependent grazers, such as African buffalo, plains zebra and blue wildebeest (Fynn et al. 2014). These outlying areas, located further away from surface water, may reduce competitive exclusion and predation risk but are typically characterised by sandy, nutrient-poor soils within open to semi-open savanna woodland. However, the sandy soils facilitate water filtration, supporting the persistence of green grass later into the dry season, while tree canopies in woodland patches support the retention of green grass frequently utilised by grazing ungulates (Treydte et al. 2008). During the dry season, sable antelope increase their consumption of grasses with low to moderate nutritional value (Hensman et al. 2014b; Owen-Smith et al. 2013), commonly found in these marginal areas. Their specialised grazing niche and ability to conserve water by producing dry faecal pellets (Woodall and Skinner 1993; Kihwele et al. 2020) likely enabled sable antelopes in our study to occupy areas farther from permanent water during the dry season, where competition and predation risk may have been reduced. However, unlike the sable antelope in northern Botswana (Hensman et al. 2014a), where occupation of the upland area was facilitated by pools that retained water into the late dry season, the dry season ranges of sable antelope in our study extended to the river.

After the ephemeral surface water dried up and evaporative cooling requirements increased with increasing heat loads during the late dry season, sable antelopes in our study travelled to and from the Kavango River, often using the same route. Travelling to water, rather than shifting between foraging patches, increased the daily distances travelled by sable antelope during the dry season (Owen-Smith and Cain 2007; Rahimi and Owen-Smith, 2007; Owen-Smith and Goodall 2014). During the dry season in northern Kruger National Park, a remnant herd of sable antelope travelled two to three times farther on days when they moved to water than on days when they did not (Cain et al. 2012). This herd travelled round-trip distances ranging 3.3–22.5 km to access distant water sources every 2–4 days during the dry season. During the late dry season, the sable antelope located farthest from the river in our study travelled approximately 13–30 km round-trip every 4–6 days to access water from the river. These travel distances to access water were longer than those reported for water-dependent plains zebra (Cain et al. 2012) and blue wildebeest (Curtin et al. 2018) and likely took time away from other activities.

The increased time spent travelling to and from the river (~ 2 h per day) coincided with a substantial reduction in time spent foraging (~ 3.5 h per day). Similarly, sable antelope in the Kruger National Park spent more time travelling during the late dry season (Owen-Smith and Goodall 2014), reducing foraging time by an average of 4 h per day on days when they moved to water (Cain et al. 2012). In contrast, when water was widely distributed within their dry season range, sable antelope showed no increase in travel time (Owen-Smith and Goodall 2014). We acknowledge that our inference that sable antelopes travelled to and from the Kavango River to drink during hot and dry conditions is based on the spatial distribution of GPS locations classified as relocating, and we were unable to confirm drinking events directly. Some long-distance movements in the relocating state may have included movements between distant foraging areas when forage quality and availability were most limited during the dry season. Likewise, increased local movement during the dry season may reflect longer distances travelled while searching for forage, including foraging while moving (i.e. a mixed movement state). Similarly, sable antelope in Kruger National Park showed increased mixed movement, mainly foraging interspersed with relocation, during the dry season (Owen-Smith and Goodall 2014). Our findings highlight how an increased reliance on evaporative cooling under high heat loads, combined with little access to preformed water when vegetation was brown, may force antelope to move further in search of water particularly during increasingly hot and dry conditions, suggesting that these specialist antelope may face trade-offs between travelling to water and searching for food as spatiotemporal variation in resource availability increases.

Increased travel to water resources may incur additional energy and time costs during an already energy-limited dry season. Despite their highly efficient muscles (Curtin et al. 2018), the long-distance movements of blue wildebeest to key resources may come at an energetic cost, as reflected in their low minimum body temperatures during the dry season (Boyers et al. 2019). Trade-offs in maintaining energy and water balance will become increasingly difficult under a hotter and drier future, especially when limited food availability (droughts) coincides with elevated water requirements to dissipate heat under high heat loads. Sable antelope, and other specialist grazers with high water dependence, may face a heightened risk of extirpation as resources become scarcer and more unpredictably distributed in space and time. While monitoring changes in seasonal and daily movements can provide early indicators of stress exposure before declines in fitness and population performance occur (Kotler et al. 2007), the conservation of antelope with specialised resource and habitat requirements depends on maintaining habitat quality, reliable access to key resources, and functional habitat heterogeneity to support adaptive resource selection in dynamic environments (Fynn et al. 2014; Fynn and Provenza 2023).

### Highlighted Student Research

Resource limitations under high heat load drive movement and behavioural trade-offs in sable antelope, demonstrating how an ecologically sensitive resource specialist navigates resource constraints.

## Supplementary Information

Below is the link to the electronic supplementary material.

Supplementary Material 1

## Data Availability

The GPS data that support the findings of this study will be shared through the AfriMove Initiative https://euromammals.org/afrimove/.

## Code Availability

The R code used for data analyses in this study is available from the corresponding author on reasonable request.
