# Supplementary material for: Vegetation greenness and heat load drive the movements of a water-dependent, selective grazer
Source: Oecologia. 2026 Jul 30;208(8):104. doi: 10.1007/s00442-026-05933-2 (PMC13424227; doi:10.1007/s00442-026-05933-2)
Supplement: Supplementary file 1 — Supplementary Material 1 [file 442_2026_5933_MOESM1_ESM.pdf]

Table S1. Linear mixed model results for the seasonal variation in home (95% isopleth) and core (50% isopleth) range size of ten free-living, female sable antelope in Bwabwata National Park, Namibia, from 1 May 2016 to 30 April 2018.

Model	β	95% CI	t	*P*
**Home range size**				
Intercept – early dry	2.324	2.147, 2.501	25.61	< 0.0001
Season – early wet	0.680	0.502, 0.854	7.42	<0.0001
Season – late dry	0.482	0.314, 0.649	5.50	<0.0001
Season – late wet	0.053	-0.129, 0.232	0.57	0.575
Year 2	-0.101	-0.249, 0.041	-1.36	0.181
**Core range size**				
Intercept – early dry	0.582	0.403, 0.761	6.31	< 0.0001
Season – early wet	0.769	0.591, 0.945	8.31	< 0.0001
Season – late dry	0.457	0.287, 0.626	5.17	< 0.0001
Season – late wet	0.109	-0.073, 0.291	1.15	0.256
Year 2	-0.124	-0.271, 0.019	-1.65	0.105

Model descriptions: log (range size) ~ season + year + (1 l individual identity), n = 10, N = 56. Model estimates (β) and 95% profile confidence intervals (95% CI) are presented on the log-transformed scale. Home and core range sizes were estimated using *a*-LoCoH. Seasons: early dry (May–July), late dry (August–October), early wet (November–January), late wet (February–April), across two years (1 May 2016 to 30 April 2018).

Haylock KA*, Parrini F, Strauss WM, Beytell P, Moeller C-H, Hetem RS
*kiara.haylock@wits.ac.za/kiara.haylock1@gmail.com

Table S2. Tukey-adjusted post hoc pairwise comparisons of seasonal differences in home (95% isopleth) and core (50% isopleth) range size in ten free-living female sable antelope in Bwabwata National Park, Namibia, from 1 May 2016 to 30 April 2018.

Contrast	β	SE	df	t	P
**Home range**					
Early dry – Early wet	-0.680	0.092	42.8	-7.40	<0.001
Early dry – Late dry	-0.482	0.088	42.2	-5.50	<0.001
Early dry – Late wet	-0.053	0.094	43.1	-0.56	0.942
Early wet – Late dry	0.198	0.093	42.7	2.13	0.159
Early wet – Late wet	0.627	0.098	42.4	6.41	<0.001
Late dry – Late wet	0.429	0.095	43.0	4.49	<0.001
**Core range**					
Early dry – Early wet	-2.006	0.252	42.8	-7.97	<0.001
Early dry – Late dry	-1.083	0.240	42.2	-4.52	<0.001
Early dry – Late wet	-0.192	0.259	43.0	-0.74	0.880
Early wet – Late dry	0.923	0.254	42.7	3.63	0.004
Early wet – Late wet	1.814	0.268	42.4	6.78	<0.001
Late dry – Late wet	0.892	0.261	42.9	3.41	0.008

Post hoc pairwise comparisons were derived from fitted linear mixed models using estimated marginal means. Models included season as a fixed effect, year retained as a non-significant control variable, and individual sable antelope identity as a random effect. Estimates (β) and standard errors (SE) represent differences between seasonal means on the log-transformed response scale. df = degrees of freedom. Home and core ranges were estimated using *a*-LoCoH. Seasons: early dry (May–July), late dry (August–October), early wet (November–January), late wet (February–April).

Haylock KA*, Parrini F, Strauss WM, Beytell P, Moeller C-H, Hetem RS
*kiara.haylock@wits.ac.za/kiara.haylock1@gmail.com

Table S3. Variance Inflation Factor (VIF) values for (a) models including all predictor variables, and (b) the best-supported models, selected using the lowest Akaike Information Criterion (AIC).

(a) Models including all predictor variables	VIF
**24-Hour mean hourly displacement distance**	
proportion of brown vegetation exposure	1.01
24-hour mean black globe temperature	3.86
24-hour maximum black globe temperature	3.84
**Proportion of 24-hour cycle in relocating state**	
proportion of brown vegetation exposure	1.02
24-hour mean black globe temperature	4.22
24-hour maximum black globe temperature	4.20
**Proportion of 24-hour cycle in foraging state**	
proportion of brown vegetation exposure	1.01
24-hour mean black globe temperature	3.71
24-hour maximum black globe temperature	3.70
**Proportion of 24-hour cycle in local movement state**	
proportion of brown vegetation exposure	1.01
24-hour mean black globe temperature	4.02
24-hour maximum black globe temperature	4.00
(b) Best supported models	
**24-Hour mean hourly displacement distance**	
proportion of brown vegetation exposure	1.01
24-hour mean black globe temperature	1.01
**Proportion of 24-hour cycle in relocating state**	
proportion of brown vegetation exposure	1.02

Haylock KA*, Parrini F, Strauss WM, Beytell P, Moeller C-H, Hetem RS
*kiara.haylock@wits.ac.za/kiara.haylock1@gmail.com

24-hour mean black globe temperature	1.01
**Proportion of 24-hour cycle in foraging state**	
proportion of brown vegetation exposure	1.01
24-hour mean black globe temperature	1.01
**Proportion of 24-hour cycle in local movement state**	
proportion of brown vegetation exposure	1.01
24-hour mean black globe temperature	1.01

Haylock KA*, Parrini F, Strauss WM, Beytell P, Moeller C-H, Hetem RS
*kiara.haylock@wits.ac.za/kiara.haylock1@gmail.com

Table S4. Candidate mixed models for the relationship between response variables: (i) 24-hour mean displacement distance and (ii) proportion of the 24-hour cycle spent in each behavioural state (relocating, foraging, local movement), and environmental covariates. Models are ranked in ascending order of Akaike's information criterion (AIC). Delta AIC (Δ_i) represents the difference between each model's AIC and the top-ranked, best-fitting model, k is the number of estimated parameters, and ω_i is the AIC weight.

Fixed effects	k	AIC	Δi	ωi
**24-Hour mean hourly displacement distance**				
proportion of brown vegetation exposure + 24-hour mean black globe temperature	5	6863.27	0.00	1.00
proportion of brown vegetation exposure + 24-hour maximum black globe temperature	5	6889.35	26.08	0.00
proportion of brown vegetation exposure	4	6978.86	115.59	0.00
24-hour mean black globe temperature	4	6997.88	134.61	0.00
24-hour maximum black globe temperature	4	7031.89	168.62	0.00
**Proportion of 24-hour cycle in relocating state**				
proportion of brown vegetation exposure + 24-hour mean black globe temperature	5	12508.82	0.00	1.00
proportion of brown vegetation exposure + 24-hour maximum black globe temperature	5	12523.12	14.30	0.00
proportion of brown vegetation exposure	4	12583.06	74.24	0.00
24-hour mean black globe temperature	4	12618.81	109.99	0.00
24-hour maximum black globe temperature	4	12640.57	131.75	0.00
**Proportion of 24-hour cycle in foraging state**				
proportion of brown vegetation exposure + 24-hour mean black globe temperature	5	20528.67	0.00	1.00
proportion of brown vegetation exposure + 24-hour maximum black globe temperature	5	20616.47	87.80	0.00

Haylock KA*, Parrini F, Strauss WM, Beytell P, Moeller C-H, Hetem RS
*kiara.haylock@wits.ac.za/kiara.haylock1@gmail.com

Fixed effects	k	AIC	Δi	ωi
proportion of brown vegetation exposure	4	20763.33	234.67	0.00
24-hour mean black globe temperature	4	20941.06	412.39	0.00
24-hour maximum black globe temperature	4	21043.77	515.10	0.00
**Proportion of 24-hour cycle in local movement state**				
proportion of brown vegetation exposure + 24-hour mean black globe temperature	5	19707.69	0.00	1.00
proportion of brown vegetation exposure + 24-hour maximum black globe temperature	5	19728.63	20.94	0.00
24-hour mean black globe temperature	4	19738.69	31.00	0.00
24-hour maximum black globe temperature	4	19763.98	56.29	0.00
proportion of brown vegetation exposure	4	19838.32	130.63	0.00

The 24-hour mean hourly displacement distance models were fitted as linear mixed models with a log-transformed response variable. For AIC-based model comparison, maximum likelihood estimation was used (i.e. restricted maximum likelihood = false). Models for the proportion of the 24-hour cycle spent in a given behavioural state were fitted as beta-binomial generalised linear mixed models with a logit link function. All models included the individual identity of sable antelope as a random effect.

Haylock KA*, Parrini F, Strauss WM, Beytell P, Moeller C-H, Hetem RS
*kiara.haylock@wits.ac.za/kiara.haylock1@gmail.com

Table S5. Akaike Information Criterion (AIC) values for 2-, 3-, and 4-state hidden Markov models (HMMs) with lognormally distributed step lengths and no turning angle. Models are ranked in ascending order of AIC.

Individual Identity	HMM Model	AIC
1963	Four-state	-31527.30
	Three-state	-31076.19
	Two-state	-29496.29
1964	Four-state	-14571.93
	Three-state	-14332.26
	Two-state	-13706.93
1965	Four-state	-9068.744
	Three-state	-8980.561
	Two-state	-8688.743
1966	Four-state	-17196.56
	Three-state	-16846.39
	Two-state	-16017.02
1967	Four-state	-30359.46
	Three-state	-29973.88
	Two-state	-28634.35
1968	Four-state	-7971.37
	Three-state	-7757.37
	Two-state	-7359.98
1969	Four-state	-32332.93
	Three-state	-31969.55

Haylock KA*, Parrini F, Strauss WM, Beytell P, Moeller C-H, Hetem RS
*kiara.haylock@wits.ac.za/kiara.haylock1@gmail.com

Individual Identity	HMM Model	AIC
	Two-state	-30708.18
1970	Four-state	-8887.04
	Three-state	-8813.05
	Two-state	-8576.72
1971	Four-state	-9427.48
	Three-state	-9243.98
	Two-state	-8653.18
1972	Four-state	-34321.09
	Three-state	-34026.51
	Two-state	-32659.81

Haylock KA*, Parrini F, Strauss WM, Beytell P, Moeller C-H, Hetem RS
*kiara.haylock@wits.ac.za/kiara.haylock1@gmail.com
